# Autocrine FGF feedback can establish distinct states of Nanog expression in pluripotent stem cells: a computational analysis

**DOI:** 10.1186/s12918-014-0112-4

**Published:** 2014-09-27

**Authors:** Dora Lakatos, Emily D Travis, Kelsey E Pierson, Jay L Vivian, Andras Czirok

**Affiliations:** Department of Biological Physics, Eotvos Lorand University, Budapest, Hungary; Division of Cancer and Developmental Biology, Department of Pathology and Laboratory Medicine, University of Kansas Medical Center, Kansas City, KS USA; Department of Anatomy and Cell Biology, University of Kansas Medical Center, Kansas City, KS USA

**Keywords:** Stem cell pluripotency, Computational model, Autocrine signaling, Transcription factors, Gene expression heterogeneity, Fibroblast growth factors, Mitogen-activated protein kinase, Regulatory network

## Abstract

**Background:**

The maintenance of stem cell pluripotency is controlled by a core cluster of transcription factors, NANOG, OCT4 and SOX2 – genes that jointly regulate each other’s expression. The expression of some of these genes, especially of *Nanog*, is heterogeneous in a population of undifferentiated stem cells in culture. Transient changes in expression levels, as well as heterogeneity of the population is not restricted to this core regulator, but involve a large number of other genes that include growth factors, transcription factors or signal transduction proteins.

**Results:**

As the molecular mechanisms behind NANOG expression heterogeneity is not yet understood, we explore by computational modeling the core transcriptional regulatory circuit and its input from autocrine FGF signals that act through the MAP kinase cascade. We argue that instead of negative feedbacks within the core NANOG-OCT4-SOX2 transcriptional regulatory circuit, autocrine signaling loops such as the Esrrb - FGF - ERK feedback considered here are likely to generate distinct sub-states within the “ON” state of the core Nanog switch. Thus, the experimentally observed fluctuations in *Nanog* transcription levels are best explained as noise-induced transitions between negative feedback-generated sub-states. We also demonstrate that ERK phosphorilation is altered and being anti-correlated with fluctuating Nanog expression – in accord with model simulations. Our modeling approach assigns an empirically testable function to the transcriptional regulators Klf4 and Esrrb, and predict differential regulation of FGF family members.

**Conclusions:**

We argue that slow fluctuations in *Nanog* expression likely reflect individual cell-specific changes in parameters of an autocrine feedback loop, such as changes in ligand capture efficiency, receptor numbers or the presence of crosstalks within the MAPK signal transduction pathway. We proposed a model that operates with binding affinities of multiple transcriptional regulators of pluripotency, and the activity of an autocrine signaling pathway. The resulting model produces varied expression levels of several components of pluripotency regulation, largely consistent with empirical observations reported previously and in this present work.

**Electronic supplementary material:**

The online version of this article (doi:10.1186/s12918-014-0112-4) contains supplementary material, which is available to authorized users.

## Background

Embryonic stem (ES) cells are pluripotent cell populations that can be induced to differentiate into a variety of cell types. Mouse ES cells are derived from the inner cell mass of the blastocyst, and their capacity to either self-renew or differentiate into cells of the three germ layers; thus this cell type is a useful model to dissect the molecular regulation behind pluripotency and differentiation [1-3]. The maintenance of stem cell pluripotency is controlled by a core cluster of transcription factors, including NANOG, OCT4 and SOX2. The molecular mechanisms by which these factors act is complex and not completely characterized; however part of their critical activity includes the joint regulation of each other’s expression [4,5]. The pluripotent status of stem cells is maintained through high expression levels of these genes, and the downregulation of these factors accompanies and is required for cell differentiation. NANOG expression levels are crucial as its forced expression is sufficient to sustain pluripotency even in the absence of extracellular signaling factors such as Leukemia Inhibiting Factor (LIF) [6,7] which are otherwise required for stem cell maintenance.

**Figure 1:**
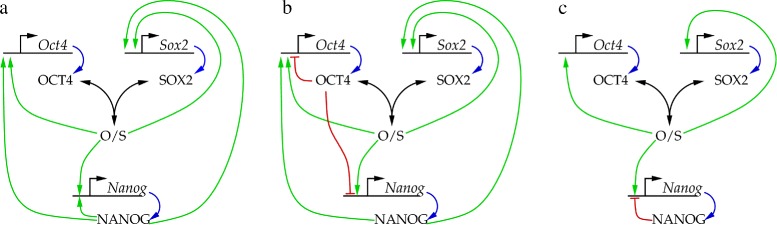
**Suggested interactions of the NANOG-OCT4-SOX2 core module of transcriptional embryonic stem cell regulation.** Black, green, red and blue arrows represent complex formation, transcriptional activation, repression and translation, respectively. **a**: The symmetric model considered by Chickarmane et al. [8]. **b**: The model suggested by Pan et al. [5] includes negative feedbacks through OCT4. **c**: The NANOG autoinhibitory circuit suggested by Navarro et al. [10].

Since the groundbreaking analysis of [8], the structure of the NANOG-OCT4-SOX2 transcriptional regulatory network has been revised – the recently proposed models are compared in Figure 1. According to the current consensus, the OCT4/SOX2 dimer acts as a common transcription factor for all three genes and no autocatalytic activation of NANOG takes place. High OCT4 levels were suggested to be repressors of NANOG (in addition to the activator function of the OCT4/SOX2 dimer) [5,9]. More recently, the OCT4 inducing function of NANOG was questioned, while NANOG was suggested to act as an autorepressor [10].

In the last few years it also become clear that – despite their fundamental importance – the expression of many components of the stem cell self-renewal circuitry, including NANOG, is heterogeneous in a population of undifferentiated stem cells [7,9,11]. The heterogeneity is dynamically maintained, with individual cells exhibiting transient changes in expression levels. Genes with dynamic expression in mouse ES cells include growth factors, transcription factors, and signal transduction proteins [10,12,13]. Recently Galvin-Burgess et al. proposed that undifferentiated ES cells are in various distinct states, depending on the activity of various signal transduction pathways [13]. The presence of states with distinct *Nanog* expression levels was also suggested based on statistical modeling of changes in flow-sorted populations [14]. Conceptually, the pluripotent and differentiating states of these cells are thus not described well by a simple “ON/OFF” switch. Instead, a cell being in one of the various pluripotent states may be primed or biased in a way that influences its response to differentiation-inducing signals [15].

**Figure 2:**
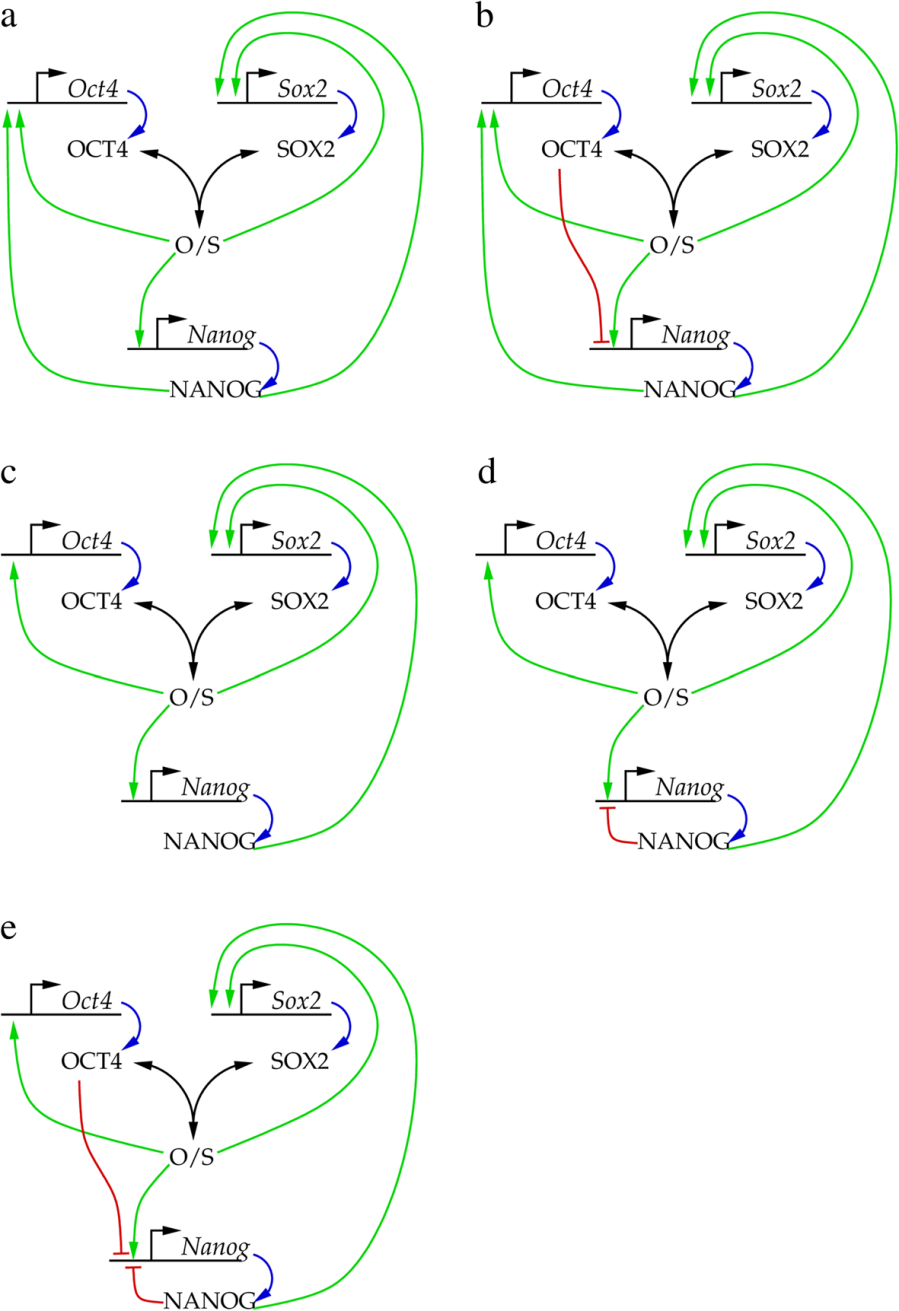
**NANOG core circuit models studied in this work.** We consider the OCT4/SOX2 dimer as a common transcription factor for all three genes, and the NANOG protein to be a transcription enhancer for the *Sox2* gene. We investigate model scenarios in which NANOG is **(a)** or is not **(c)** an inducer of *Oct4*. Furthermore, we consider further variants, such as the model proposed by Pan et al. where high OCT4 levels repress *Nanog* **(b)**, as well as the NANOG autorepressor feedback proposed by [10] **(d)** and a combination of NANOG autorepressor feedback with OCT4 repression **(e)**.

In view of these developments, we revisit the dynamics of the core NANOG transcriptional regulatory circuit. As shown in Figure 2, we will consider the OCT4/SOX2 dimer as a common transcription factor for all three genes, and the NANOG protein to be a transcription enhancer for the SOX2 gene. We consider four model scenarios, in which NANOG either is or is not an inducer of OCT4. We consider the models proposed by Pan et al. where high OCT4 levels are repressors of NANOG and OCT4 [5], and that of Navarro et al., which includes an autorepressor feedback to NANOG [10]. By numerical simulations we demonstrate that all these models result in a bistable, switch-like behavior. To address the observed heterogeneity in NANOG expression levels, we also explore a biologically plausible scenario to couple the core circuit to extracellular signals. Based on simulation results we argue that instead of an instability within the core regulatory circuit, fluctuations in NANOG expression levels and associated distinct cell states are likely to be generated by stochastic autocrine feedback loops, like the one involving secreted FGFs.

## Results

### Model structure

To explore the NANOG transcriptional regulatory network, we adopted the method of [8]. The production and degradation of proteins are assumed to be much slower than the assembly or dissociation of multimolecular complexes, we thus include the latter processes using a quasi steady state approximation. With these simplifications the system is governed by three differential equations of the form

(1) $$ (1 + D_{\mathrm{G}}) \frac{d\!\left[\mathrm{G}\right]}{dt} = \alpha_{g} p_{g} - \delta_{\mathrm{G}} \!\left[\mathrm{G}\right]  $$

where G=NANOG,OCT4,SOX2 are transcription factor proteins, *g* denotes the regulatory site of a gene *G*, *p*_*g*_ is the probability of RNA Polymerase II (P) binding to the promoter *g*; *α*_*g*_ is the combined translation and transcription rate, and *δ*_G_ is the decay rate of the proteins. The quasi steady state approximation yields the amount of complex-bound specimen, [G_*bound*_] as a function of the concentration of free specimen [G]. As a change in the total amount alters both the amount of free and complex-bound specimen,

(2) $$ d\!\left[\mathrm{G}\right] + d\!\left[\mathrm{G}_{bound}\right] = d\!\left[\mathrm{G}\right] \left(1 + \frac{\partial \!\left[\mathrm{G}_{bound}\right]}{ \partial\!\left[\mathrm{G}\right]} \right).  $$

Thus,

(3) $$ D_{\mathrm{G}}=\frac{\partial \!\left[\mathrm{G}_{bound}\right]}{ \partial\! \left[\mathrm{G}\right]}.   $$

As we show in the Additional file 1, *p*_*g*_ can be written in the form of

(4) $$ p_{g} = \frac{Z^{ON}_{g}}{ Z_{g}},   $$

where

(5) $$ Z_{g}=Z^{ON}_{g} + Z^{OFF}_{g}  $$

and the $Z^{ON}_{g}$ and $Z^{OFF}_{g}$ quantities are proportional to the probability of RNA polymerase II being bound or absent at locus *g*, respectively. If the transcription logic is limited to two factors (enhancers or repressors) per locus, then for each locus *g*∈{*Nanog*,*Oct4*,*Sox2*} we obtain

(6) $$ {\fontsize{9}{12}\begin{aligned} Z^{ON}_{g} &= \left[\mathrm{P}\right]\! K_{g, \mathrm{P}} \left[ 1 + \sum\limits_{{\substack{\mathrm{H}\in\{\textrm{NANOG}, \\ \text{OS},\textrm{OCT4}\}}}} \left[\mathrm{H}\right]\! K_{g,\mathrm{H}} \exp{\left(\frac{C_{g,\mathrm{H}} }{ RT} \right)} + \right.\\ &\qquad\qquad\;\;\;\;\left.+ \sum\limits_{{\substack{\mathrm{H},\mathrm{I}\in\{\textrm{NANOG}, \\ \text{OS},\textrm{OCT4}\}}}} \left[\mathrm{H}\right]\! K_{g,\mathrm{H}} \!\left[\mathrm{I}\right]\! K_{g,\mathrm{I}} \exp{\left(\frac{C_{g,\mathrm{H},\mathrm{I}} }{ RT} \right)} \right]  \end{aligned}}  $$

and

(7) $$ Z^{OFF}_{g} = 1 + \!\!\sum\limits_{{\substack{\mathrm{H}\in\{\textrm{NANOG}, \\ \text{OS},\textrm{OCT4}\}}}} \left[\mathrm{H}\right]\! K_{g,\mathrm{H}} + \!\! \sum\limits_{{\substack{\mathrm{H},\mathrm{I}\in\{\textrm{NANOG}, \\ \text{OS},\textrm{OCT4}\}}}} \!\! \left[\mathrm{H}\right]\! K_{g,\mathrm{H}} \!\left[\mathrm{I}\right]\! K_{g,\mathrm{I}}.   $$

In the expressions (6) and (7) the equilibrium constant (binding affinity) of factor H to the binding site at locus *g* is denoted by

(8) $$ K_{g,\mathrm{H}}=\frac{\left[g_{bound}\right]}{\left[g_{empty}\right]\!\left[\mathrm{H}\right]}.  $$

As we discuss in the Additional file 1, the equilibrium constants *K* as well as the cooperativity measures *C* are related to the binding energies between the transcription factors, the promoter and the RNA polymerase.

The magnitude of model parameters (Additional file 1: Tables S1 and S2) were set by the following considerations. The transcription and translation rates were chosen in such a way that the steady state transcription factor (protein) concentrations are in the nanomolar range (in the order of 100 copy of the TF is present in the cell) when the promoter is fully active [16,17]. To get a functional transcriptional regulatory system, the nanomolar concentration range must be also characteristic for promoter binding affinities, which by Additional file 1: Eq. (S1) translates (at *T*=300 K) into binding energies around 12 kCal/mol. The transcription factors were assumed to work through stabilizing RNAP II binding – with protein-protein binding energies around 4 kCal/mol [16]. This binding energy is increased for cooperative, multimolecular complexes. We assume that the probability of RNAP II binding in the absence of all the transcription factors considered is very low, [*P*] *K*_*g*,P_≈10^−3^.

### The core network

First we consider various scenarios for the core NANOG circuit (Figure 2) and compare their behavior. Steady state system behavior was characterized by numerically obtaining intersections of nullcline planes (see Additional file 1: Figure S1).

Our starting point is the model A, which is symmetric in the roles of SOX2 and OCT4 (Figure 2a). This model exhibits bistability: there are two stable fixed points corresponding to the “ON” and “OFF” states of the system, separated by an unstable fixed point. Linear stability analysis reveals that the stable fixed points are stable nodes, thus, no oscillations are expected in their vicinity.

**Figure 3:**
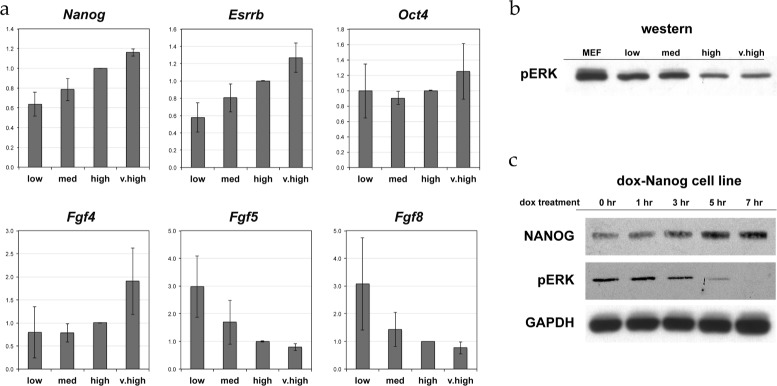
**The behavior of various signaling components during NANOG fluctuations.** **a**: Expression levels, as determined by rt-pcr, of NANOG, SOX2, OCT4, ESRRB and the FGFs active within mouse ESCs. Data show fold change differences in expression normalized to Gapdh transcript levels. Each expression value is the average of values obtained from three independent experiments. **b**: ERK activity in cell populations with various extent of NANOG expression. As a positive control, we also include pERK western blot data from mouse embryonic fibroblasts (MEF). **c**: Western analysis of dox-inducible Nanog ES cell line. Note reduction of pERK levels in response to increasing amounts of NANOG.

Augmenting the model with a negative feedback through OCT4, as suggested by [5,9], can be accomplished by increasing the binding affinity of the OCT4 protein to the *Nanog* regulatory site, and decreasing the stability of the OCT4-containing RNAP II complex (Figure 2b). We assume that the binding affinity of OCT4 is *lower* than that of NANOG or the OCT4/SOX2 dimer – reflecting that high concentration of OCT4 (overexpression) was needed to elicit the inhibition. Once OCT4 is bound, however, we assume a strong inhibitory effect. As suggested [9], this change indeed can transform the “ON” state from a stable node to a stable spiral, but only if the OCT4 binding affinity is *higher* than the values characteristic for the other TFs. In such a case the fluctuations in [OCT4] are of similar magnitude than that of [NANOG] (data not shown). As OCT4 levels appear quite stable in mouse embryonic stem cells ([13], Figure 3), in models compatible with this observation the direct OCT4 negative feedback is unlikely to play an important role.

In model C (Figure 2c), OCT4 is independent of NANOG activation. In such a scenario the same parameters that were used for model A yield only the “OFF” fixed point as *OCT4* never turns on. Considering the steady presence of OCT4 in embryonic stem cells, we argue that for this model it is reasonable to choose an increased probability for RNAP II binding to the *Oct4* locus even in the absence of SOX2. This choice yields a bistable system similar to that of model A.

Model D (Figure 2d) is derived from model C by adding a NANOG autorepression feedback. As Additional file 1: Figure S1d demonstrates, this change does not alter substantially the systems dynamics as the Nanog promoter activity can be obtained by scaling Additional file 1: Eq. (S17) the activity of model C.

Finally, we combine model D with model B to see if NANOG autorepression can further promote the transformation of the “ON” fixed point into a spiral. Adding NANOG autorepression to model B reduces the equilibrium NANOG levels (as determined by the amount of OCT4 present) according to Additional file 1: Eq. (S17), which further stabilizes the fixed point. We also derived model E (Figure 2e) by adding OCT4 as a *Nanog* repressor to model D. In this scenario we found the fixed point still strongly attractive: oscillations decay fast and change both NANOG and OCT4 levels to a similar extent.

This analysis of the core NANOG circuit variants suggest that they are likely to behave as bistable systems. Hence, the experimentally seen heterogeneity, given the stability of OCT4 expression levels ([13], Figure 3), is an unlikely consequence of the core NANOG-OCT4-SOX2 dynamics. In the following we will focus on model D, as the simplest variant of the investigated networks, and functionally equivalent with the one proposed by the most recent experimental data [10]. This choice, however, is somewhat arbitrary as all model networks function as a bistable switch.

### Sensitivity of the core network to model parameters

Changes in model parameter values can gradually shift the nullclines and fixed points in the phase space. As changes in nullcline positions and shapes can create or remove intersections, the presence of both the “OFF” and “ON” states are parameter dependent.

**Figure 4:**
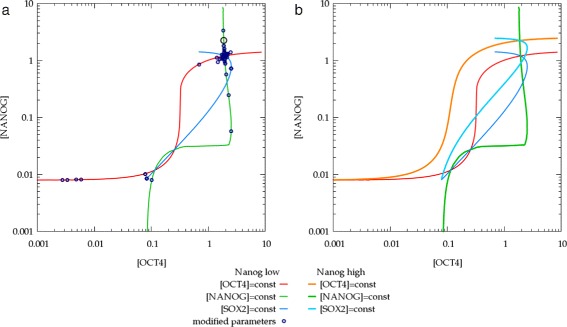
**Parameter sensitivity analysis of the “ON” fixed point of the core NANOG circuit.** **a**: Each model parameter was changed by 20 and 40% and the obtained new steady states (blue circles) are overlayed on the phase space plot of Additional file 1: Figure S1d. Of particular interest is a change in *C*_*Nanog*,OS_, the increase of binding energy of RNAP II at the *Nanog* promoter in the presence of the OCT4/SOX2 dimer. Twenty percent change in this parameter shifts the equilibrium NANOG concentration by more than an order of magnitude more than that of OCT4 (black circle). **b**: Parameter dependence of nullclines and fixed points: the orange and cyan curves were obtained in a simulation with increased *C*_*Nanog*,OS_. Notice that in the “ON” state, NANOG concentration is increased by a factor of 2, while OCT4 levels remained the same. Furthermore, the altered set of parameters excludes the “OFF” state of the system. Concentrations are presented in the units of nM.

To gauge the model’s sensitivity to parameter values, we systematically varied all of them, one-by-one, by 20% and 40%. Starting the simulations from the “ON” fixed point, we obtained the new steady state values under the altered parameter setting. As Figure 4 demonstrates, most parameters effect only one molecular species directly, and the fixed point moves along a corresponding nullcline intersection line. In particular, if the “ON” fixed point falls onto the saturated regime of the nullcline intersections, then the steady state concentrations of model components, such as [NANOG] and [OCT4] may be differently altered. For example, changing the binding energy between RNAP II and the OCT4/SOX2 dimer at the *Nanog* promoter by 20% shifts the equilibrium NANOG concentration by more than an order of magnitude more than that of OCT4. This observation is the basis of our explanation for NANOG heterogeneity and OCT4 homogeneity within a cell population.

### NANOG heterogeneity

As we demonstrated above, the most plausible assumptions do not suggest the presence of substantial oscillations within the core NANOG-OCT4-SOX2 system. To explain the observed broad distribution of NANOG expression within a population of mouse ES cells, we hypothesize that model parameters such as binding energies depend on the larger biochemical context. For example, ERK activity is a known potent negative regulator of NANOG transcription [12,13]. In turn, FGF signaling is capable to create an autocrine feedback using cell surface receptors that feed into the MAPK pathway, and FGF activity is indeed a well established modulator of ES cell heterogeneity [18]. Nodal signaling gives an alternative possibility for an autocrine extracellular regulation of Nanog, mediated through the Smad family of transcription factors [13].

To demonstrate that autocrine signaling loops can induce fluctuating Nanog expression levels, we consider a feedback through FGFs and ERK. As a particular example, we investigate a scenario in which the stability of the RNAP II complex containing the OCT4/SOX2 dimer depends on ERK activation. To explore the behavior of the FGF pathway during NANOG fluctuations, mouse ES cells were sorted based on their NANOG expression levels into four groups (low, medium, high, very high). For each group we determined the transcriptional activity of key genes, such as OCT4, ESRRB, and FGF family members expressed by mouse ES cells: FGF4, FGF5 and FGF8. As Figure 3 demonstrates, the range of Oct4 variability is less than half of that of Nanog. In contrast, the range of variability in the expression levels of FGFs and ESRRB is even greater than that of NANOG. High FGF4 expression is associated with high Nanog expression, whereas high FGF5 and FGF8 expression is characteristic for cells with low levels of Nanog. ERK activity (phosphorilation) assays revealed an inverse relationship between ERK activity and Nanog expression levels (Figure 3). This correlation between Nanog expression and ERK activity is seen in both sorted Nanog subpopulations (Figure 3b) and in a doxycycline-inducible Nanog ES cell line (Figure 3c).

**Figure 5:**
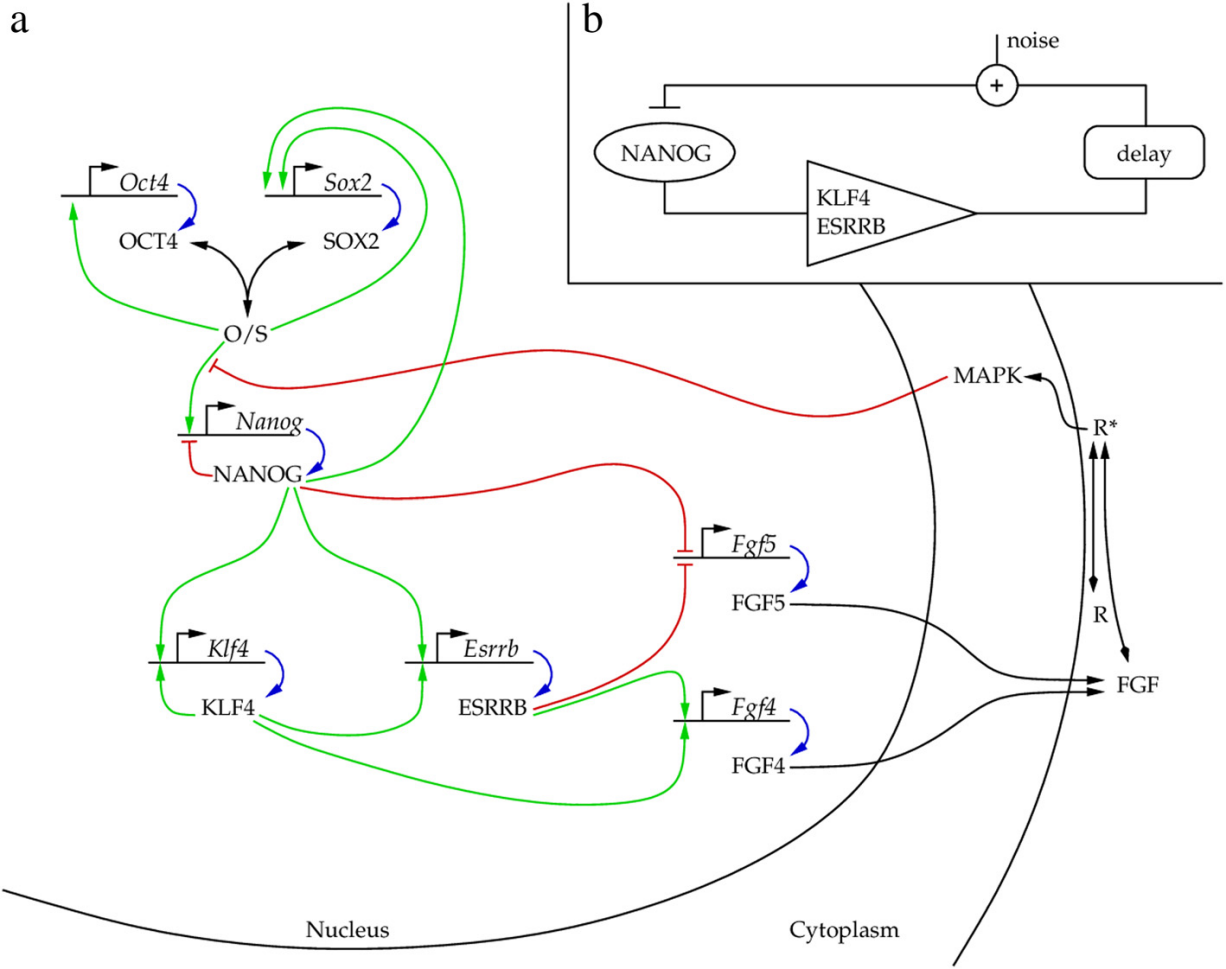
**The signaling and transcriptional network considered to regulate mouse ES cell maintenance.** **(a)** Full model. Green, red and blue arrows represent transcriptional activation, repression and translation, respectively. Black arrows represent complex formation, and various multi-step processes: (i) FGF secretion resulting in an effective autocrine ligand concentration and (ii) activation of the MAPK. **(b)** Schematic representation of the autocrine feedback loop. The suggested model acts as a noisy negative feedback regulator which includes a signal amplifier and delay.

Based on known regulatory binding sites [19], and the expression data in Figure 3, we consider the autocrine feedback loop shown in Figure 5. Using the available transcription factor-DNA binding ChiP data set, we selected two transcriptional regulators for each gene. In particular, we assume that the KLF4-ESRRB system is downstream of the NANOG core circuit. We suggest that NANOG, KLF4 and ESRRB are activators and repressors of the two FGF genes considered in the model. Finally, we assume that secreted FGF proteins bind to cell surface receptors in an autocrine manner. The activation of FGF receptors initiate the intracellular MAPK signaling pathway [20], which closes the feedback to NANOG through modulating the binding affinities of the OCT4/SOX2 dimer, the only transcription factor of NANOG explicitly considered in the model. In the following we build up this complex signaling model from simpler modules.

### KLF4 and ESRRB

**Figure 6:**
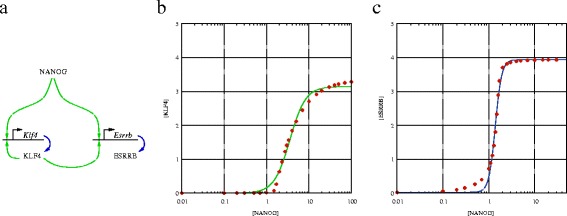
**The KLF4-ESRRB module as an amplifier.** We consider an autocatalytic regulation of KLF4, and cooperative positive regulation from NANOG **(a)**. Keeping [NANOG] at various pre-determined values, we obtained the steady state concentrations and promoter activity of KLF4 and ESRRB. The relationship between these values and [NANOG] is strongly non-linear, reflecting the autoregulation and cooperative binding with NANOG. The solid line represent fitted Hill curves with *n*=2.3±0.2 and *n*=6.2±0.4 for KLF4 and ESRRB, respectively **(b, c)**. Concentrations are presented in the units of nM.

*Klf4* and *Esrrb* are known to be pluripotency genes and as a recent study exposed, both are direct targets of NANOG [21]. KLF4 was reported to bind to it’s respective promoter as well as to the promoter regions of *Nanog* and *Esrrb* [22]. To keep our model as simple as possible, we restrict the number of regulatory connections to two per locus. We further assume that ESRRB is more downstream than KLF4 is (see Figure 6a). Promoter binding affinities were chosen in such a way that [NANOG] is in the nanomolar range when *Klf4* and *Esrrb* genes switch on. With such assumptions, the NANOG-KLF4-ESRRB cascade can function as an amplifier. By keeping [NANOG] steady (as input), the autocatalytic expression levels of KLF4 are well approximated by a Hill function of exponent 2 (Figure 6b). The steady state expression level of ESRRB is an even more non-linear function of [NANOG]: the abrupt switch is steeper than a Hill function with *n*=6 (Figure 6c). Thus, consistent with empirical data, if the KLF4-ESRRB system is tuned in this regime of operation, changes in the transcriptional activity of ESRRB may exceed by an order of magnitude that of NANOG.

### Autocrine FGF signaling

Chromatin immunoprecipitation assays revealed that the *Fgf4* gene has binding sites for KLF4 and ESRRB, and the *Fgf5* gene has sites for NANOG and ESRRB [23,24]. Microarray expression analysis [25] has shown that FGF Receptor 1, that can bind all three flavors of secreted FGFs, is expressed by mouse ES cells. Thus, we work with an aggregate autocrine FGF concentration, [FGF^∗^] to determine downstream receptor activity. As FGF5 and FGF8 expression levels appear to be similarly regulated, in our model both are represented by FGF5. While the dynamics of autocrine FGF signaling has not been studied in mouse embryonic stem cells, autocrine EGF signaling was explored extensively in other experimental systems [26]. Experiments with autocrine EGF signals revealed a linear relationship between cell surface autocrine ligand concentration and the production rate of the protein. Thus, we assume that the equation governing autocrine FGF ligand concentration, [FGF^∗^], is

(9) $$ \frac{d\!\left[\textrm{FGF}^{*}\right]}{ dt} = \sum\limits_{i\in\{4,5\}} \frac{\alpha_{\textit{Fgf-} i} p_{\textit{Fgf-} i} - \delta_{\textrm{FGF-} i} \!\left[\textrm{FGF-} i\right]}{1 + D_{\textrm{FGF-} i}}  $$

where 0<*p*_*Fgf-i*_<1 is the probability of transcription at the *Fgf4* and the *Fgf5* locus, and *δ*_FGF-*i*_ is the decay rate combined with the diffusive flux transporting the ligand off the cell surface. The *α*_*Fgf-i*_ coefficient reflects both production and the conversion between autocrine ligand concentration and production. In the EGF system the relationship between steady state autocrine ligand concentration and its production rate was 0.05 pM/(molecules/cell/h) ≈ 0.05 pM/(30 pM/h) ≈ 0.1% h (see Figure six of [26]). Based on this result, we expect that less than 1% of the FGF molecules produced in an hour (our approximate time unit) will act as autocrine ligands at the cell surface. Therefore, providing autocrine ligand concentration in the nanomolar range requires higher production rates than the rates we assumed for transcription factors (see Additional file 1: Table S1).

The MAPK cascade, the signaling pathway downstream of the FGF receptor, has been studied extensively both by computational and biochemical methods [27-29]. These studies revealed two characteristic operation mode: changes in receptor ligation may elicit a transient and a sustained ERK activity. Interestingly, both responses are well approximated by a linear response function. Since the characteristic lifetime of the transient response is in the order of minutes, we assume that the relatively slow changes in *Nanog* expression that take place over several hours reflect a sustained change in steady state ERK activation. Our experimental data (Figure 3) is also consistent with a change in the steady state ERK activity. Hence, we propose that the normalized difference

(10) $$ \varepsilon = \frac{\left[\textrm{ERK}^{*}\right] - \left[\textrm{ERK}^{*}\right]_{0}} {\left[\textrm{ERK}^{*}\right]_{0}}  $$

between ERK activity (i.e., concentration of phospho-ERK, [ERK^∗^]) and a reference (baseline) level activity [ERK^∗^]_0_ is proportional to the number of active receptor complexes *R*^∗^ as

(11) $$ \varepsilon \sim R^{*}.   $$

The steady state concentration of active receptors is

(12) $$ R^{*}=R^{tot}\frac{\left[\textrm{FGF}^{*}\right]}{1/K + \left[\textrm{FGF}^{*}\right]},  $$

where *R*^*tot*^ is the total amount of FGF receptors at the cell surface, and *K* is the binding constant between the receptors and their FGF ligands.

The connection between ERK activity and NANOG activation is currently unknown. Here we assume, that the regulation involves the modulation of the binding affinity of the OCT4/SOX2 dimer, the only transcription factor of NANOG that is explicitly considered in the model:

(13) $$ C_{\textit{Nanog},\text{OS}} - C_{\textit{Nanog},\text{OS}}^{(0)} \sim \varepsilon,  $$

thus

(14) $$ C_{\textit{Nanog},\text{OS}} - C_{\textit{Nanog},\text{OS}}^{(0)} = a R^{tot} \frac{\left[\textrm{FGF}^{*}\right]} {1/K + \left[\textrm{FGF}^{*}\right]}   $$

where the coupling factor *a* is chosen in such a way that for a typical simulation the magnitude of (14) is smaller than one.

### NANOG expression determined by autocrine feedback

**Figure 7:**
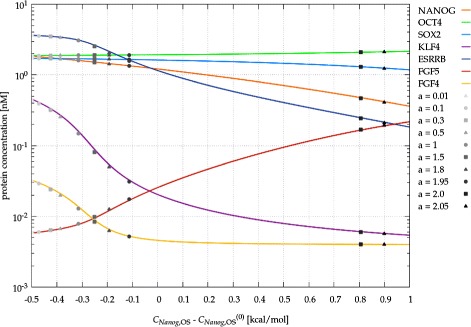
**The effects of modulating the transcriptional regulation of** ***Nanog*** **.** Steady state protein concentrations are plotted for various values of *C*_*Nanog*,OS_, the binding energy among RNAP II, the OCT4/SOX2 dimer and the *Nanog* locus. If the binding energy is set by ERK activity through the feedback (14), the system evolves into a steady state (gray symbols) which depends on parameter *a*, the strength of the feedback.

**Figure 8:**
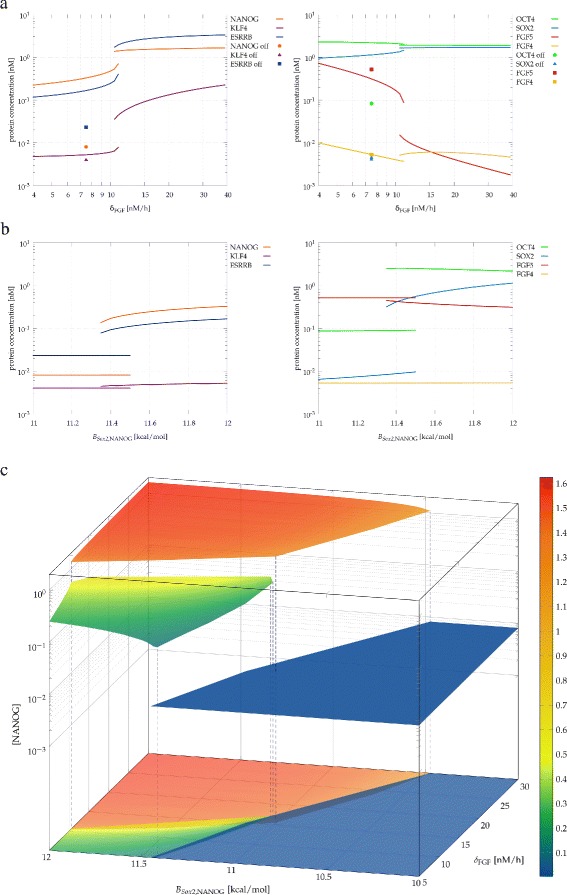
**Presence of substates within the “ON” state of the core** ***Nanog*** **switch.** Steady state protein concentrations obtained for strong autocrine feedback (*a*=2) as a function of the autocrine ligand decay parameter *δ*_FGF_ which is sensitive to the efficiency of autocrine ligand capture **(a)**. The abrupt change and the hysteresis indicates the existence of distinct sub-states. Both sub-states are within the “ON” state of the core NANOG circuit as the parameters identified in Figure 4, like *B*_*Sox2*,NANOG_, the the binding energy of NANOG to the *Sox2* promoter, are still able to shut off *Nanog* expression (solid symbols). The switch between the “ON” and “OFF” states continues to involve a bifurcation as the sudden jumps and hysteresis indicates **(b)**. The “ON” and “OFF” states as well as the two substates can be visualized in a three dimensional parameter space, where the steady state NANOG concentration is plotted as a function of the the autocrine ligand decay parameter *δ*_FGF_, and the binding energy of NANOG at the *Sox2* locus, *B*_*Sox2*,NANOG_ **(c)**. Concentrations are presented in the units of nM.

To explore the behavior of the full model (Figure 5), we first consider steady states obtained with various (fixed) values of *C*_*Nanog*,OS_, the binding energy between RNAP II and the OCT4/SOX2 dimer at the *Nanog* promoter – the assumed site of ERK regulation (Figure 7). When *C*_*Nanog*,OS_ is set by the feedback (14) with a specific “gain” parameter *a*, the system reaches a single steady state that falls on the steady state curves shown in Figure 7. We propose that the observed Nanog heterogeneity is resulted by slow alterations in model parameters – specific for individual cells – like the feedback strength or the efficiency of autocrine ligand capture. For strong enough autocrine feedbacks the system is characterized by a fold in the phase space (pitchfork bifurcation), hence small changes in the parameter values can have disproportionally large effects on the steady state concentration values. Furthermore, due to the hysteresis distinct sub-states can co-exist (Figure 8), hence the concentration values also reflect the history of the system. All these sub-states are, however, still within the “ON” state of the NANOG core circuit: the parameters identified in Figure 4, like the the binding affinity of NANOG to the *Sox2* promoter, are still able to shut off *Nanog* expression through another sudden change (Figure 8c). To address the robustness of the substates, we performed a systematic variation of model components by 10%. We found, that when both distinct “ON” and “OFF” states were present, the “ON” state exhibited substates separated by a bistable region in 85% of the cases.

**Figure 9:**
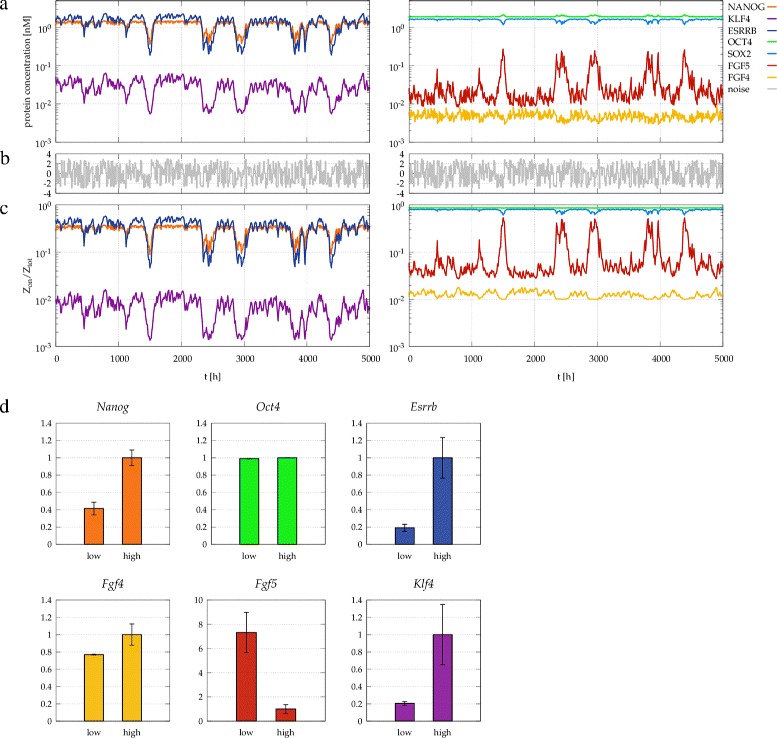
**Fluctuations in protein concentrations and gene expression driven by a noise.** Fluctuations in protein concentrations **(a)** and gene expression **(c)**, driven by a noise **(b)** added to the autocrine ligand decay parameter *δ*_FGF_, which changes its value by ±30*%*. In the time series regimes with high and low NANOG concentration were identified, using threshold concentrations of 1.0 nM and 0.7 nM for NANOG high and low expression, respectively. The corresponding time-averaged expression levels are plotted in panel **(d)**.

Large fluctuations readily develop as a response to a high frequency noise added to the parameters. As an example, a 30% modulation of the FGF decay parameter *δ*_FGF_ results in slow, but large amplitude transitions between the substates (Figure 9a-c): the model-predicted duration of a transition is in the order of a day, and the typical time of the system spends in the same substate is in the order of a week. Such simulations also allow to correlate expression levels in the model with experimental data shown in Figure 3. In the time series we identified regimes where [NANOG] was below 0.7 nM (“Nanog low”) or above 1.0 nM (“Nanog high”). Fow both types of time intervals we averaged the expression level of each factor, and normalized it to the “Nanog high” state (Figure 9d). The general tendencies of both Figure 9d and Figure 3 are identical: *Nanog* expression levels are more variable than that of *Oct4*, and the KLF4-ESRRB amplifier can further increase the variability in *Fgf* expression.

## Discussion

### The mechanism behind Nanog fluctuations

Protein and mRNA levels can fluctuate due to the inherently stochastic kinetics of gene transcription and translation. Even an unregulated, constitutively expressed gene exhibits a 30% spread of expression levels over a population of identical cells [30]. Clearly, the reported variations in the stem cell maintenance network, especially those genes that are downstream of *Nanog*, greatly exceed this baseline variability. Thus, we expect that the observed heterogeneity in Nanog expression levels is generated by the dynamics of the regulatory system as it greatly amplifies the molecular stochastic noise. To describe a potential mechanism generating the experimentally observed dynamics, we proposed a model that operates with binding affinities of multiple transcriptional regulators of pluripotency, the topology of the transcriptional regulatory networks, and activity of an autocrine signaling pathway. The resulting model produces varied expression levels of several components of pluripotency regulation, largely consistent with our (Figure 3) and previously reported empirical observations [13].

We argue that slow fluctuations in *Nanog* expression likely reflect individual cell-specific changes in parameters of an autocrine feedback loop, such as changes in ligand capture efficiency, receptor numbers or the presence of crosstalks within the MAPK signal transduction pathway. While high-frequency variability may be filtered out by the slow dynamics of transcription factor synthesis and accumulation, low frequency changes, such as a slow alteration in the cell’s microenvironment, are capable to push expression levels across substate boundaries. Given the complexities of a cell’s variable exposure to autocrine/juxtacrine signaling in culture, this model incorporates a plausible basis for a variable activity of an autocrine signaling pathway eliciting heterogeneous expression of intracellular components. In this view the fluctuations of Nanog reflect the response of a regulatory system with multiple feedbacks in a non-stationary environment.

This feedback mechanism, that does not involve changes in OCT4, is consistent with both the stability of *Oct4* expression levels [13], (Figure 3) and the observation that alteration of OCT4 levels induces ES cells to differentiate rapidly; too much or too little OCT4 rapidly directs cells to differentiate [31,32]. Very small increases in OCT4 expression causes differentiation to mesoderm and endoderm, and reduction of OCT4 levels induces loss of pluripotency and dedifferentiation to trophectoderm.

### Comparison with experimental data

The proposed computational model needs to be compared with experimental data on at least three levels. (1) The dynamics of the whole system can be evaluated and compared with corresponding experimental data. (2) Our model makes explicit or implicit assumptions on the topology of the transcriptional regulatory network, and identifies functional modules. Finally, (3) most model parameters are expressed either as molecular binding affinities or as parameters effecting the stability of the transcription complex in addition to production and decay rates.

#### Dynamics

Our model calculations demonstrate that a biologically plausible autocrine feedback can create distinct sub-states within the “ON” state of the core Nanog switch. While stochasticity and feedback regulation has been proposed to explain Nanog fluctuations [9,17,33], the previously proposed mechanisms involved noise induced transitions between the “ON” and “OFF” states of the core *Nanog* switch. Experimental evidence, however, suggests that *Nanog* expression in *Nanog*-low ES cells is still much higher than that in cells committed to differentiation [13]. Furthermore, *Nanog*-low cells can still be maintained indefinitely without committing to differentiation – their Nanog expression level will, in fact, increase. These empirical observations clearly support a mechanism that operates with transitions between substates that do not involve the “OFF” state of the core *Nanog* switch. The existence of distinct substates of Nanog expression was also suggested by a recent study that analyzed experimentally observed changes in Nanog expression profiles in terms of a phenomenological model that made no explicit assumptions on the underlying molecular signaling network [14].

Our approach also demonstrates that the slow modulation of Nanog expression can reflect changes that are external to the core circuit – instead of the stochastic expression of the transcription factors [17]. The topology of the regulatory network considered here is more elaborated than in previous studies [9,17], and thus able to represent and predict changes in expression of several downstream genes. Furthermore, our model does not include direct autoregulation of Nanog – a frequent assumption in simplified models which lacks empirical support.

FGF and NODAL signaling are clearly active in mouse embryonic stem cells, and function in an autocrine fashion in undifferentiated cells [18]. Previous studies [12,13] have shown that these autocrine signaling pathways influence the dynamic heterogeneity of ES cells in culture, likely through specific molecular mechanisms that have yet to be elaborated. Our results show highly variable expression of FGF ligands and intracellular ERK activity in ES cells grown in serum-based media (Figure 3). The model behavior presented in Figure 9, is largely consistent with the anticorrelation found in ERK activity and Nanog expression (Figure 3) as well as existing information on FGF signaling [12].

#### Regulatory network topology

The regulatory network shown in Figure 5 contains motifs that are well established as well as hypothetical regulatory connections that we explore in this work. The full network can be broken up into four modules: the core NANOG switch, the downstream ESRRB amplifier, the autocrine FGF module and finally, the feedback through the MAPK cascade.

Our analysis indicates that each of the recently proposed regulatory architecture of the core NANOG-OCT4-SOX2 network (Figure 2) functions as a bistable switch. We argue, that irrespective of the underlying model details, the nonlinearity needed to create two stable fixed points is provided by the autoregulation and the dimerization of the OCT4/SOX2 transcription factors. The switch-like behavior is also maintained when OCT4 acts as a likely low affinity repressor – an assumption motivated by the finding that OCT4 overexpression was required for the manifestation of the repressive behavior [34].

The roles KLF4 and ESRRB play in stem cell maintenance are in the focus of recent scientific interest [21,35,36]. Based on chromatin immunoprecipitation data [19], both the *Klf4* and *Esrrb* genes have several potential regulatory sites, which include transcription factors from the Nanog core as well as allow autoregulatory feedbacks. We demonstrated that a subset of the known likely regulatory interactions is capable to function as an amplifier, greatly expanding the variability of these factors. While the existence of (functional) autoregulation in these loci has not yet been established by targeted experiments, the proposed amplifier function of the KLF4-ESRRB system is in good agreement with the experimentally obtained large variations in *Esrrb* (Figure 3 and [37]) and downstream *Fgf* expression levels (Figure 3).

The *Fgf* genes which are known to be actively transcribed in mouse ES cells exhibit regulatory sites for the transcription factors considered in this work. Chromatin immunoprecipitation data reveals that *Fgf4* has putative regulatory sites for KLF4 and ESRRB, *Fgf5* has putative regulatory sites for NANOG and ESRRB, while *Fgf8* exhibits a regulatory site for NANOG [19]. While the nature of the regulatory connection is currently unknown, based on the strong correlation between *Nanog* and *Fgf4* expression and the strong anticorrelation between *Nanog* and *Fgf5* as well as *Nanog* and *Fgf8* expression levels, we assume that *Fgf5* and *Fgf8* are repressed while *Fgf4* expression is enhanced by the transcription factors considered in this work. The repressional regulation of *Fgf* genes is of key importance – it yields a negative feedback loop, and this will tested by targeted experiments in the future.

Finally, the MAPK module and the autocrine ligand concentration was treated phenomenologically by simplified input-output relationships reflecting previously established results. In particular, we postulated a linear relationship between autocrine ligand concentration and its rate of production [26,38]. The MAPK pathway can exhibit transient and sustained activation [39,40], the latter lasting only for less than an hour [28]. As changes in transcriptional regulation are operating on a slower time scale, here we considered a change in sustained ERK activation, which was also carefully studied and is expected to operate as an amplifier (ultrasensitive switch) linking input and output with a Hill coefficient much greater than one [27,41]. This observation motivates our use of a coupling factor *a* in Eq. (14) that is able to substantially reduce *Nanog* expression, in agreement with specific observations in mouse ES cells [12,13].

#### Parameter values

A molecular regulatory network model is bound to have several parameters, for most of which very little empirical data is available. As one way to contain this problem, here we used a modular approach: first we identified the behavior of smaller units, like the core Nanog switch, or the Esrrb amplifier. Functional requirements can considerably constrain the possible parameter values within such a module. As a second effort to reduce the arbitrariness of model parameters we derived expression rates from binding energies [16,42]. Finally, unless empirical evidence suggested otherwise, we kept the simplest (uniformly assigned) values of the parameters and did not try to match empirical data by fine tuning the parameters. Nevertheless, we do not consider our parameters to be predictive – many different combinations can give similar overall behavior. Yet, they are important to demonstrate that the proposed regulatory system can work with plausible parameter values.

### Future directions

The major focus of this model involves a regulatory network of Nanog, Oct4, and Sox2; these transcriptional regulators constitute a semi-independent regulatory module (‘core’ module [43]) in maintaining pluripotency. Clearly many other transcriptional regulators are involved in this complex module however. Our model represents an extensible platform for adding further components of the core module. The integration of other transcriptional regulators such as Tcf3 [44], Tbx3 [45], and signaling pathways such as Nodal and BMP signaling [13], will be an important future application of this model to study the inherent instability of the pluripotent phenotype in serum-based media. The model presented here does not take into account potential role of allelic expression of the *Nanog* locus [46] in directing heterogeneity in Nanog expression. Experimental data for this phenomenon are contradictory, however, [47]. Further experimental data will be required to confirm this phenomenon, and if so, how this unusual mode of transcriptional regulation may be integrated into the model presented here.

## Conclusions

The pluripotency of embryonic stem cells is sustained by a core cluster of co-regulated transcription factors, NANOG, OCT4 and SOX2. Surprisingly, Nanog as well as several other downstream transcription factors exhibit widely fluctuating expression in a population of undifferentiated stem cells. To explain the observed heterogeneity of expression levels, we propose a computational model that couples the transcriptional regulation of Nanog to autocrine extracellular signals. We argue that the likely source of fluctuations is not the core regulatory cluster, but stochasticity within the autocrine feedback loops. The model predicts fluctuating expression levels for several factors involved in pluripotency maintenance, largely consistent with empirical observations presented here or reported previously. Our model indicates the presence of distinct substates of pluripotency, each exhibiting various expression levels of Nanog and downstream transcription or signaling factors. Thus, we predict that subpopulations within undifferentiated embryonic stem cells can have non-uniform responses to extracellular stimuli. Finally, we assign an empirically testable function to the transcriptional regulators Klf4 and Esrrb, and predict differential regulation of FGF family members. In vivo, we expect autocrine feedbacks to be highly sensitive to alterations of the stem cell microenvironment, hence our model can guide future studies linking stem cell maintenance to physical and biochemical properties of the stem cell niche.

## Methods

### Mouse ES cell culture

Experiments used the BAC-Nanog-GFP (BNG) ES cells [13] in which *Nanog*-GFP bacterial artificial chromosome (BAC) [48] was introduced into Ainv15 ES cells [49]. BNG ES cells were maintained as described previously [13,25] on gelatin-coated or fibroblast cocultured plates. Cells were grown in serum-based ES cell media: Dulbecco’s modified Eagle’s medium, 15% fetal bovine serum (FBS), penicillin-streptomycin, L-glutamine, nonessential amino acids, *β*-mercaptoethanol, and 10^3^ units/ml leukemia inhibitory factor (LIF).

### Fluorescent cell sorting and analysis

ES cells were trypsinized to a single cell suspension and analyzed by BD FACSAria (cell sorting) and BD LSRII flow cytometer (cell analysis) for GFP expression. A convention was established for sorting and analyzing subpopulations of BNG ES cells based on the profile of GFP expression of unsorted cells, as described in [13]. The GFP-medium and GFP-high populations were determined by gating 30%–35% of the cells from the peak of the GFP distribution. Cells expressing higher levels of GFP than the GFP-high subpopulation were classified as GFP-very high; whereas cells with expression less than GFP-medium cells but above background were identified as GFP-low cells. Analysis of control E14 ES cells were used as a negative control for flow analysis and sorting. After cell sorting, subpopulations were analyzed to confirm purity of the population.

### RNA analysis

RNA was isolated (Qiagen, Hilden, Germany), and cDNA was synthesized (Invitrogen) following manufacturer’s instructions. TaqMan primer sets with the 7500 Real Time polymerase chain reaction (PCR) system (Applied BioSystems, Foster City, CA) were used for quantitative real-time PCR analysis. Upon request, specific ABI TaqMan Primer/Probe assay identification numbers are available.

### Protein analysis

Cells were pelleted and then lysed with radioimmune precipitation assay buffer supplemented with Halt Protease and Phosphatase Inhibitor Cocktails (Pierce, Rockford, IL). Protein samples were separated on BioRad Tris-HCl gels, and blots were probed with primary antibodies for GAPDH (Santa Cruz Biotechnology, Santa Cruz, CA) and pERK (Cell Signaling) and incubated overnight at 4 deg C with appropriate secondary antibodies. SuperSignal West Pico Chemiluminescence (Pierce) was used to detect the Western blots.

### Dox-inducible Nanog ES cell line

The Nanog coding sequence was inserted into a dox-responsive locus via CRE-mediated insertion using previously described methods [13,25,49] into the AInv-15 ES cell line, to generate BNG-dox-Nanog cell line. These cells were cultured under feeder-free conditions in standard serum based ES cell media supplemented with doxycycline (1 ug/ml) for the indicated times. Cells were then harvested in RIPA buffer for western analysis.

## Additional file

Additional file 1
**Supplemental information.** Supplement: detailed description of the model structure, analysis of core networks; **Tables S1–S3.** parameter values used in model simulations.
