# Supplementary material for: Autocrine FGF feedback can establish distinct states of Nanog expression in pluripotent stem cells: a computational analysis
Source: BMC Syst Biol. 2014 Sep 27;8:112. doi: 10.1186/s12918-014-0112-4 (PMC4189679; doi:10.1186/s12918-014-0112-4)
Supplement: Additional file 1 — Supplemental information. Supplement: detailed description of the model structure, analysis of core networks; Tables S1–S3. parameter values used in model simulations. [file 12918_2014_112_MOESM1_ESM.pdf]

Supplement

Promoter activity

Let us denote the Gibbs free energy of binding between a transcriptional regulator H and the corresponding regulatory site of gene g by $B_{g,\mathrm{H}}$. This quantity is related to the equilibrium constant $K_{g,\mathrm{H}}$ as

$$RT \ln K_{g,\mathrm{H}} c_0 = B_{g,\mathrm{H}} \tag{S1}$$

where c_0 is the standard concentration (the dimension of K being M^{-1}).

Following [1, 2], gene transcription activity is controlled by the binding probability of RNAP II (P), which, in turn is determined by the binding energy of the assembled complex at the promoter and regulatory sites. The binding energy of the complex, in the presence of a transcriptional regulator, H, is $E_{g,\mathrm{H}}$. It is convenient to express this quantity as

$$E_{g,\mathrm{H}} = B_{g,\mathrm{P}} + B_{g,\mathrm{H}} + C_{g,\mathrm{H}}, \tag{S2}$$

where the "cooperativity" C measures the difference between the binding energy of the complex and the binding energies of the individual constituents to the DNA sequence (in the absence of any other factors). In the simplest case $C_{g,\mathrm{H}}$ represents a "direct" binding energy between the transcription factor and RNAP II, but it can also reflect conformation changes induced by the presence of H. Similarly, in the case of a RNAP II complex containing two transcription factors, H and I, the binding energy can be written as

$$E_{g,\mathrm{H,I}} = B_{g,\mathrm{P}} + B_{g,\mathrm{H}} + B_{g,\mathrm{I}} + C_{g,\mathrm{H,I}} \tag{S3}$$

The concentration or probability of a particular complex can be derived from the binding energies by the generalization of Eq. (S1). For example, the concentration of the four component complex consisting the binding site g, RNAP II and factors H and I is

$$[complex] = [g][\mathrm{H}][\mathrm{I}][\mathrm{P}]K_{g,\mathrm{H,I,P}} = \frac{[g][\mathrm{H}][\mathrm{I}][\mathrm{P}]}{c_0^3} \exp\left(\frac{E_{g,\mathrm{H,I}}}{RT}\right), \tag{S4}$$

where $[g]$,[H],[I] and [P] denotes the (time averaged) steady state concentration of the regulatory site g without any of the other model components bound to it, the concentrations of free transcription factors H and I and the concentration of unbound RNAP II, respectively.

Promoter binding parameters

To evaluate the promoter activity (Eq. (4)), the binding energies B and the C parameters characterizing cooperativity needs to be specified. As these values are not characterized in the literature, the following assumptions are made.

If transcription factor binding affinities are in the nanomolar range, Eq. (S1) predicts a typical value of $B = 12$ kCal/mol. If intramolecular bonds between the components of the RNAP II complex is the main contributor to its enhanced stability, then a rough estimate for the C parameters is 2 kCal/mol for each pair of physical interaction [2], thus $C = 4$ kCal/mol for two transcription factors that each interact with the RNAP II but do not bind each other directly. Repression of transcription activity can be represented by large negative C values, which destabilizes the repressor-containing complex.

In the absence of any transcriptional regulator, the promoter activity is

$$p_g^{(0)} = \frac{[\mathrm{P}]K_{g,\mathrm{P}}}{1 + [\mathrm{P}]K_{g,\mathrm{P}}}. \tag{S5}$$

Thus the value of $[\mathrm{P}]K_{g,\mathrm{P}}$ can be estimated as the transcriptional activity without the considered transcription factors present. Knowing that in the absence of NANOG, OCT4 and SOX2 protein the transcription of these genes shuts down, we assume $p_g^{(0)} \approx [\mathrm{P}]K_{g,\mathrm{P}} = 10^{-3}$. While these considerations set the magnitude of the parameters, functional considerations – such as the presence of both an "ON" and an "OFF" state in the core NANOG-OCT4-SOX2 network, or the ability to model a repressor – led us to modify these values in certain cases.

Quasi Steady State Approximation (QSSA)

If the free transcription factor concentration is [H], we need to determine the amount of molecules bound to regulatory sites. As a given factor may contribute to multiple complexes, we need to take into account each. If a certain complex A contains $n_{A,\mathrm{H}}$ molecules of specimen H, and the concentration of this complex is $[A]$, then the total amount of protein bound at regulatory sites is

$$[\mathrm{H}_{bound}] = \sum_g \sum_{A \in g} n_{A,\mathrm{H}}[A], \tag{S6}$$

where the summation goes over all possible promoter sites (g) and regulatory complexes A that can form at a given promoter site as indicated by the symbolic summation rule $A \in g$. If a particular complex A, assembled at promoter g, consists of proteins H, ... I, then according to Eq. (S4)

$$[A] = [g]w_A \tag{S7}$$

where

$$w_A = \frac{[\mathrm{H}]}{c_0} \dots \frac{[\mathrm{I}]}{c_0} \exp\left(\frac{E_A}{RT}\right). \tag{S8}$$

As the total promoter concentration of locus g is $c^* \approx 1/\text{cell} \approx 0.1$ nM,

$$c^* = [g] + \sum_{A \in g}[A] = [g]\left(1 + \sum_{A \in g} w_A\right) = [g]Z_g. \tag{S9}$$

Thus,

$$[A] = \frac{c^*}{Z_g} w_A. \tag{S10}$$

Using these notations, Eq. (3) can be calculated as

$$D_\mathrm{H} = \frac{\partial[\mathrm{H}_{bound}]}{\partial[\mathrm{H}]} = \sum_g \sum_{A \in g} n_{A,\mathrm{H}} \frac{\partial[A]}{\partial[\mathrm{H}]} = c^* \sum_g \sum_{A \in g} \frac{n_{A,\mathrm{H}}}{Z_g}\left(\frac{\partial w_A}{\partial[\mathrm{H}]} - \frac{w_A}{Z_g}\frac{\partial Z_g}{\partial[\mathrm{H}]}\right) \tag{S11}$$

As w_A is a polynomial of H,

$$\frac{\partial w_A}{\partial[\mathrm{H}]} = n_{A,\mathrm{H}} \frac{w_A}{[\mathrm{H}]} \tag{S12}$$

and

$$\frac{\partial Z_g}{\partial[\mathrm{H}]} = \sum_{B\in g} n_{B,\mathrm{H}} \frac{w_B}{[\mathrm{H}]}. \tag{S13}$$

Thus, introducing the sums

$$S^{(k)}_{g,\mathrm{H}} = \sum_{B\in g} n^k_{B,\mathrm{H}} \frac{w_B}{[\mathrm{H}]}, \tag{S14}$$

which are readily evaluated knowing the free specimen concentrations [H], equation (S11) can be written as

$$\begin{aligned} D_{\mathrm{H}} &= c^* \sum_g \sum_{A\in g} \frac{n_{A,\mathrm{H}}}{Z_g} \left(n_{A,\mathrm{H}} \frac{w_A}{[\mathrm{H}]} - \frac{w_A}{Z_g} \sum_B n_{B,\mathrm{H}} \frac{w_B}{[\mathrm{H}]} \right) = \\ &= c^* \sum_g \left(\frac{S^{(2)}_{g,\mathrm{H}}}{Z_g} - [\mathrm{H}] \left(\frac{S^{(1)}_{g,\mathrm{H}}}{Z_g} \right)^2 \right) \end{aligned} \tag{S15}$$

If each complex A can contain specimen H only once, then $S^{(2)}_{g,\mathrm{H}} = S^{(1)}_{g,\mathrm{H}}$.

If a transcription factor can also form a complex in addition to the one formed at gene regulatory sites, such as the dimerization of SOX2 and OCT4, then the QSSA needs to take that into account as well. In particular, assuming equilibrium, the dimer concentration [OS] is given as

$$[\mathrm{OS}] = K_{\mathrm{OS}}[\mathrm{SOX2}][\mathrm{OCT4}]. \tag{S16}$$

Thus D_{OCT4} contains an additional term, $K_{\mathrm{OS}}[\mathrm{SOX2}]$, to those listed in (S15).

Analysis of core networks

Steady state system behavior was characterized by numerically obtaining intersections of nullcline planes – steady state concentrations obtained when one of the specimen was kept at a fixed value. For example, by keeping [NANOG] at a pre-determined value, the steady state concentration values satisfy Eq. (1) for both $g =$ *Sox2* and *Oct4* so that the corresponding time derivatives are zero. Thus the obtained concentration values are at the intersection of the $d[\mathrm{SOX2}]/dt = 0$ and $d[\mathrm{OCT4}]/dt = 0$ nullclines.

Model A As the nullcline intersections demonstrate in Fig. S1a, steady state [NANOG] is a monotonous increasing function of both [OCT4] and [SOX2] when either of these quantities are used as fixed control parameters. The [NANOG] vs [OCT4] and [NANOG] vs [SOX2] curves reflect the gradual activation of the *Nanog* promoter and the saturation of transcription at transcription factor (TF) concentrations exceeding the promoter's binding affinity. The markedly nonlinear functional form is due to the need of TF dimerization to activate transcription. Fig. S1a also demonstrates that steady state [OCT4] and [SOX2] levels are decreasing for large concentrations

of the complementary species as formation of the OCT4/SOX2 complex depletes the pool of free proteins. This simple model exhibits bistability: there are two stable fixed points corresponding to the 'ON' and 'OFF' states of the system, separated by an unstable fixed point. Linear stability analysis reveals that the stable fixed points are stable nodes, thus, no oscillations are expected in their vicinity.

Model B A negative feedback through OCT4, as suggested by [3, 4], can be accomplished by increasing the binding affinity of the OCT4 protein to the *Nanog* regulatory site, and decreasing the stability of the OCT4-containing RNAP II complex. Fig. S1b reveals that such an inhibition renders the [NANOG] vs [OCT4] curve decreasing for OCT4 concentrations higher than its binding affinity. As suggested [4], this change indeed can transform the "ON" state from a stable node to a stable spiral, but only if the OCT4 binding affinity is *higher* than the values characteristic for the other TFs. In such a case the fluctuations in [OCT4] are of similar magnitude than that of [NANOG] (data not shown).

Model C To reflect the increased probability for RNAP II binding to the *Oct4* locus, in Fig. S1c for the *Oct4* promoter we use the value $[\mathrm{P}]K_{Oct4,\mathrm{P}} = 0.02$ instead of the value 0.001 – used for all other promoters. With this choice the intersection of the OCT4 and SOX2 nullclines moves to higher OCT4 values, and yields a bistable system where two stable nodes are separated by an unstable fixed point. Since in this model OCT4 production only depends on the dimer concentration, [OCT4/SOX2], the [OCT4] vs [SOX2] curve is the same irrespective if we set [SOX2] directly or indirectly through [NANOG].

Model D As Fig. S1d demonstrates, a NANOG autorepression feedback does not alter substantially the systems dynamics: when *Nanog* is turned on, the steady state NANOG concentrations are somewhat reduced compared to the values of model C (gray lines). Potent autorepression does not change Z_{Nanog}^{ON} as $\exp(C_{Nanog,\mathrm{NANOG}}) = \exp(C_{Nanog,\mathrm{NANOG,OS}}) \approx 0$. In contrast, autorepression increases Z_{Nanog}^{OFF} by $[\mathrm{NANOG}]K_{Nanog,\mathrm{NANOG}}$. Thus the probability of transcription in the models with or without autorepression, p'_{Nanog} and p_{Nanog}, respectively, can be directly compared by a suitable scaling as

$$p'_{Nanog} \approx p_{Nanog} \frac{1}{1 + [\mathrm{NANOG}]K_{Nanog,\mathrm{NANOG}} Z_{Nanog}}. \tag{S17}$$

Model E Adding OCT4 as a *Nanog* repressor to model D renders the [NANOG] vs [OCT4] curve decreasing for OCT4 concentrations higher than its binding affinity. The fixed point remains strongly attractive: oscillations decay fast and change both NANOG and OCT levels to a similar extent.

References

1. Shea, M.A., Ackers, G.K.: The or control system of bacteriophage lambda. a physical-chemical model for gene regulation. J Mol Biol **181**(2), 211–230 (1985)
2. Buchler, N.E., Gerland, U., Hwa, T.: On schemes of combinatorial transcription logic. Proc Natl Acad Sci U S A **100**(9), 5136–5141 (2003)
3. Pan, G., Thomson, J.A.: Nanog and transcriptional networks in embryonic stem cell pluripotency. Cell Res **17**(1), 42–49 (2007)
4. Kalmar, T., Lim, C., Hayward, P., Muñoz-Descalzo, S., Nichols, J., Garcia-Ojalvo, J., Arias, A.M.: Regulated fluctuations in nanog expression mediate cell fate decisions in embryonic stem cells. PLoS Biol **7**(7), 1000149 (2009)

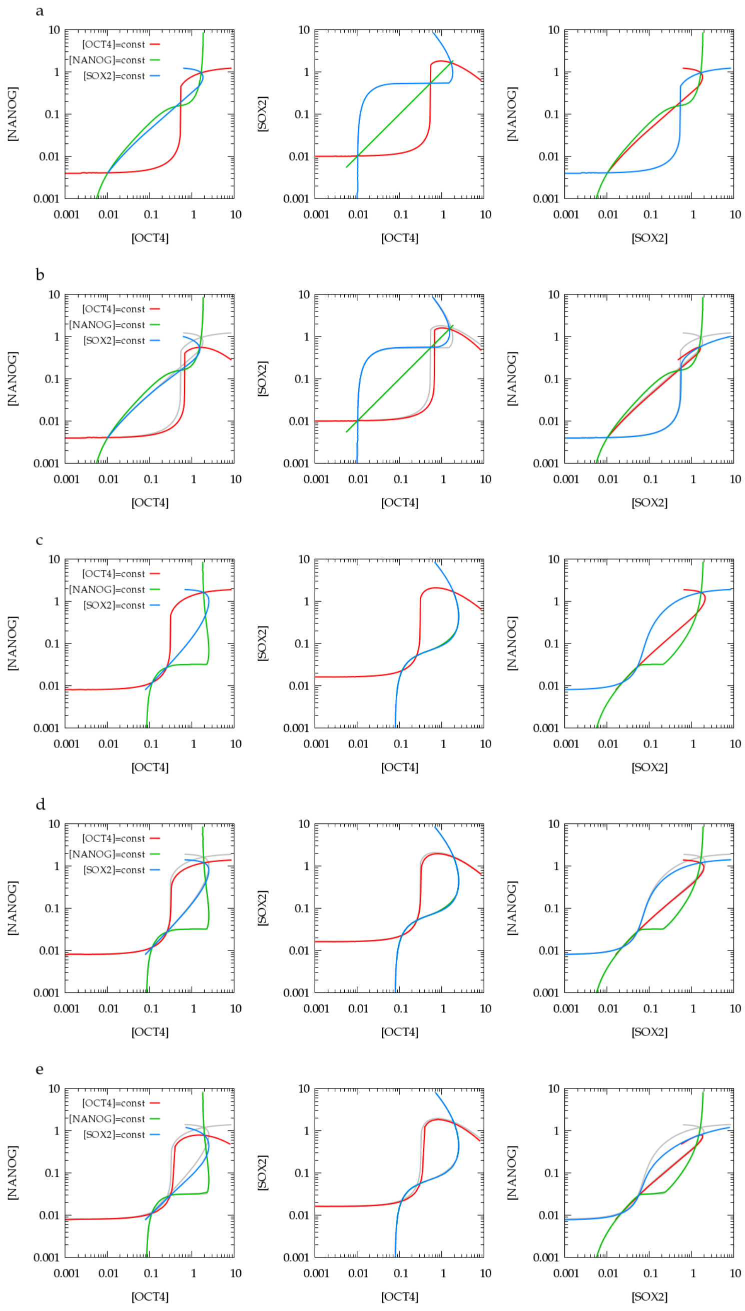

Figure S1 Steady state properties of the NANOG core circuit model variants shown in Fig. 2. Each panel is annotated as the corresponding model variant (A-E). Steady state concentrations were obtained in simulations where one of the molecular specimen was kept at a fixed value (NANOG: green, OCT4: red and SOX2: blue). Intersection of all three curves indicate fixed points. Two of these fixed points, located at low and high concentrations are stable nodes in models A, C and D: a perturbed system is expected to return to these states without oscillations. In model B and E the fixed point at high concentrations may turn into a stable spiral. In contrast, the fixed point at intermediate concentration is unstable. Thus, the system behaves as a bistable switch, with distinct “ON” and “OFF” states. To ease comparison between model variants, nullclines of models A, C and D are plotted with gray lines in panels b, d and e, respectively. Concentrations are presented in nM units.

Tables

Table S1 Model parameters I: binding energy and three-component cooperativity. $B_{g,\mathrm{TF}}\left[\frac{\mathrm{kcal}}{\mathrm{mol}}\right]$: binding energy of TF at locus g; $C_{g,\mathrm{TF}}\left[\frac{\mathrm{kcal}}{\mathrm{mol}}\right]$: cooperativity among RNAP II, TF and locus g.

	$B_{g,\mathrm{TF}} \mid C_{g,\mathrm{TF}}$					
gene\TF	NANOG	OCT4	OS	SOX2	KLF4	ESRRB
Nanog	12 | -99	0 | 0	12 | 4	0 | 0	0 | 0	0 | 0
Oct4	0 | 0	0 | 0	12 | 4	0 | 0	0 | 0	0 | 0
Sox2	12 | 4	0 | 0	12 | 4	0 | 0	0 | 0	0 | 0
Klf4	11 | 0	0 | 0	0 | 0	0 | 0	11 | 0	0 | 0
Esrrb	12 | 0	0 | 0	0 | 0	0 | 0	12 | 0	0 | 0
Fgf5	14 | 0	0 | 0	0 | 0	0 | 0	0 | 0	15 | 0
Fgf4	11 | 0	0 | 0	0 | 0	0 | 0	11 | 0	0 | 0

Table S2 Model Parameters II: four-component cooperativity and RNAP II binding. $C_{g,\mathrm{TF}_1,\mathrm{TF}_2}\left[\frac{\mathrm{kcal}}{\mathrm{mol}}\right]$: cooperativity among RNAP II, TF_1, TF_2 and locus g, where $B_{g,\mathrm{TF}_1}, B_{g,\mathrm{TF}_2} \neq 0$; $K_{g,\mathrm{P}}\left[\frac{1}{\mathrm{nM}}\right]$: binding probability of RNAP II (P) at locus g. [1] The probability for RNAP II binding to the Oct4 locus was set to 0.001 in model variant A and B.

genes	$C_{g,\mathrm{TF}_1,\mathrm{TF}_2}$	$[\mathrm{P}]K_{g,\mathrm{P}}$
Nanog	-99	0.002
Oct4		0.02[1]
Sox2	6	0.001
Klf4	6	0.001
Esrrb	6	0.005
Fgf5	-99	100
Fgf4	4	0.01

Table S3 Model Parameters III

translation and transcription rate	α_{gene}	$\left[\frac{\mathrm{nM}}{\mathrm{h}}\right]$	4
decay rates for TFs	δ_{TF}	$\left[\frac{\mathrm{nM}}{\mathrm{h}}\right]$	1
autocrine decay rates	δ_{FGF}	$\left[\frac{\mathrm{nM}}{\mathrm{h}}\right]$	10
OCT4/SOX2 association rate	k_a	$\left[\frac{1}{\mathrm{nMh}}\right]$	1
OCT4/SOX2 dissociation rate	k_d	$\left[\frac{1}{\mathrm{h}}\right]$	1
FGF receptor concentration	R^{tot}	$[\mathrm{nM}]$	1
Receptor ligand binding energy	$B_{\mathrm{R,L}}$	$\left[\frac{\mathrm{kcal}}{\mathrm{mol}}\right]$	11
Indirect inhibition rate	a	$\left[\frac{\mathrm{kcal}}{\mathrm{mol}}\frac{1}{\mathrm{nM}}\right]$	10
